# Aphids harbouring different endosymbionts exhibit differences in cuticular hydrocarbon profiles that can be recognized by ant mutualists

**DOI:** 10.1038/s41598-021-98098-2

**Published:** 2021-10-01

**Authors:** Corinne Hertaeg, Marion Risse, Christoph Vorburger, Consuelo M. De Moraes, Mark C. Mescher

**Affiliations:** 1 Department of Environmental Systems Science, ETH Zürich, Zürich, Switzerland; 2 Department of Aquatic Ecology, Eawag, Swiss Federal Institute of Aquatic Science and Technology, Dübendorf, Switzerland

**Keywords:** Ecology, Chemical ecology

## Abstract

Cuticular hydrocarbons (CHCs) have important communicative functions for ants, which use CHC profiles to recognize mutualistic aphid partners. Aphid endosymbionts can influence the quality of their hosts as ant mutualists, via effects on honeydew composition, and might also affect CHC profiles, suggesting that ants could potentially use CHC cues to discriminate among aphid lines harbouring different endosymbionts. We explored how several strains of *Hamiltonella defensa* and *Regiella insecticola* influence the CHC profiles of host aphids (*Aphis fabae*) and the ability of aphid-tending ants (*Lasius niger*) to distinguish the profiles of aphids hosting different endosymbionts. We found significant compositional differences between the CHCs of aphids with different infections. Some endosymbionts changed the proportions of odd-chain linear alkanes, while others changed primarily methyl-branched compounds, which may be particularly important for communication. Behavioural assays, in which we trained ants to associate CHC profiles of endosymbiont infected or uninfected aphids with food rewards, revealed that ants readily learned to distinguish differences in aphid CHC profiles associated with variation in endosymbiont strains. While previous work has documented endosymbiont effects on aphid interactions with antagonists, the current findings support the hypothesis that endosymbionts also alter traits that influence communicative interactions with ant mutualists.

## Introduction

The insect cuticle contains long-chain hydrocarbons that aid in water retention and serve as a physical barrier against pathogens and parasites^1,2^. These cuticular hydrocarbons (CHCs) also have important intraspecific communicative functions, which have been especially well-studied in social insects, where they play crucial roles in nestmate recognition, colony organization and the coordination of foraging activities^3^. The significance of CHCs for interspecific communication is less clear, although several studies have shown that ants tending myrmecophilous aphids use CHCs to recognize suitable aphid partners^4–8^, which they protect from predators and parasitoids, while collecting aphid honeydew^9^. One factor that might be expected to influence both honeydew quality^10^ and the CHC composition of the aphid cuticle is the presence of facultative endosymbiotic bacteria, which have previously been shown to influence many ecologically relevant traits of their hosts^11^. However, the potential significance of endosymbiont mediated effects on aphid CHC profiles for interactions with aphid-tending ants (or other organisms) has not previously been explored.

While all aphids exhibit an obligate association with the primary endosymbiont *Buchnera aphidicola*, which synthesizes essential amino acids that are missing in the aphid diet^12^, they also form facultative associations with a number of other endosymbionts. Some of these have been shown to provide ecologically important benefits to aphids, including increased heat tolerance^13,14^, enhanced performance on specific host plants^15^, protection against pathogenic fungi^16,17^, and reduced susceptibility to parasitoid wasps^18,19^. However, association with secondary endosymbionts has also been found to have adverse effects on aphids, including reduced competitive ability, as well as reduced longevity and fecundity^14,20–23^. Such adverse effects may be explained in part by competition for resources between the host and its endosymbionts, which frequently exhibit reduced metabolic capacities and are heavily reliant on host nutrients^24,25^. In particular, as the phloem sap diet of aphids is poor in nutrients other than carbohydrates, endosymbionts may frequently be in competition with hosts for amino acids and lipids^10,26,27^.

In addition to direct impacts on aphids, such competition might also influence ecological interactions between aphids and other organisms, including those mediated by CHCs. Our current understanding of the biosynthesis and composition of aphid CHCs remains limited; however, lipid metabolism is closely linked to the biosynthesis of CHCs^3,28^ and amino acids such as isoleucine and valine are precursors for common methyl-branched CHCs^29^. Furthermore, there is evidence from a handful of non-aphid insect species that endosymbionts can affect host CHC profiles^30–34^, although the relevant studies focused primarily on non-communicative functions of CHCs (e.g., in desiccation resistance) or on specific CHC compounds that function as sex pheromones. Competition for nutrients or other impacts of endosymbionts on host metabolism might also influence the quality of aphid honeydew, which plays a key role in mutualistic interactions with aphid-tending ants. For example, reduced concentrations of amino acids in the honeydew of endosymbiont-infected aphids compared to uninfected ones have been reported^10^. It is therefore plausible that different secondary endosymbionts could have variable effects on the quality of honeydew for aphid mutualists while simultaneously inducing changes in CHC profiles that might enable ants to reliably discriminate between aphids harbouring different endosymbionts.

Previous work has shown that aphid-tending ants use CHC profiles to recognize suitable myrmecophilous aphid partners^4–6,8^. This recognition mechanism may be particularly important during the earliest stages of aphid-ant association, when direct assessment of honeydew quality may not be a reliable indicator because myrmecophilous aphids frequently increase the nutritional quality of their honeydew only after ants begin tending them^35–37^. Aphids presumably also benefit from being recognized as good partners, both in order to reap the benefits of the mutualism and because ants selectively prey on aphids depending on their nutritional requirements^38,39^. The presence of aphids with different endosymbiont infections^10^, as well as different aphid clones within species^40^, may present ants with potential mutualists of differing quality that may also exhibit different CHC profiles, raising the question of whether ants can distinguish between aphids infected with different endosymbionts based on the aphid CHC profiles.

To address this question, we set out to investigate whether the CHC profiles of *Aphis fabae* aphids are altered by infections with different endosymbiotic bacteria and whether an ant mutualist (*Lasius niger*) can perceive these differences. In addition to their ecological significance, aphids represent highly tractable systems in which to explore such questions because of their clonal reproduction and our ability to manipulate symbiont communities via microinjection. In the current study, we inoculated each of two, previously uninfected, clonal aphid lines with five different strains of two common bacterial endosymbionts, *Hamiltonella defensa* (three strains) and *Regiella insecticola* (two strains)^41^ and analysed the CHC profiles of inoculated aphids and uninfected controls. To determine whether the resulting differences in CHC profiles between aphids harbouring different endosymbionts were perceptible by ants, we then conducted behavioural trials testing whether *L. niger* workers could learn to associate relevant CHC variation with a honey reward.

## Results

### Facultative endosymbionts alter the composition of aphid cuticular hydrocarbons in clone-dependent ways

**Figure 1:**
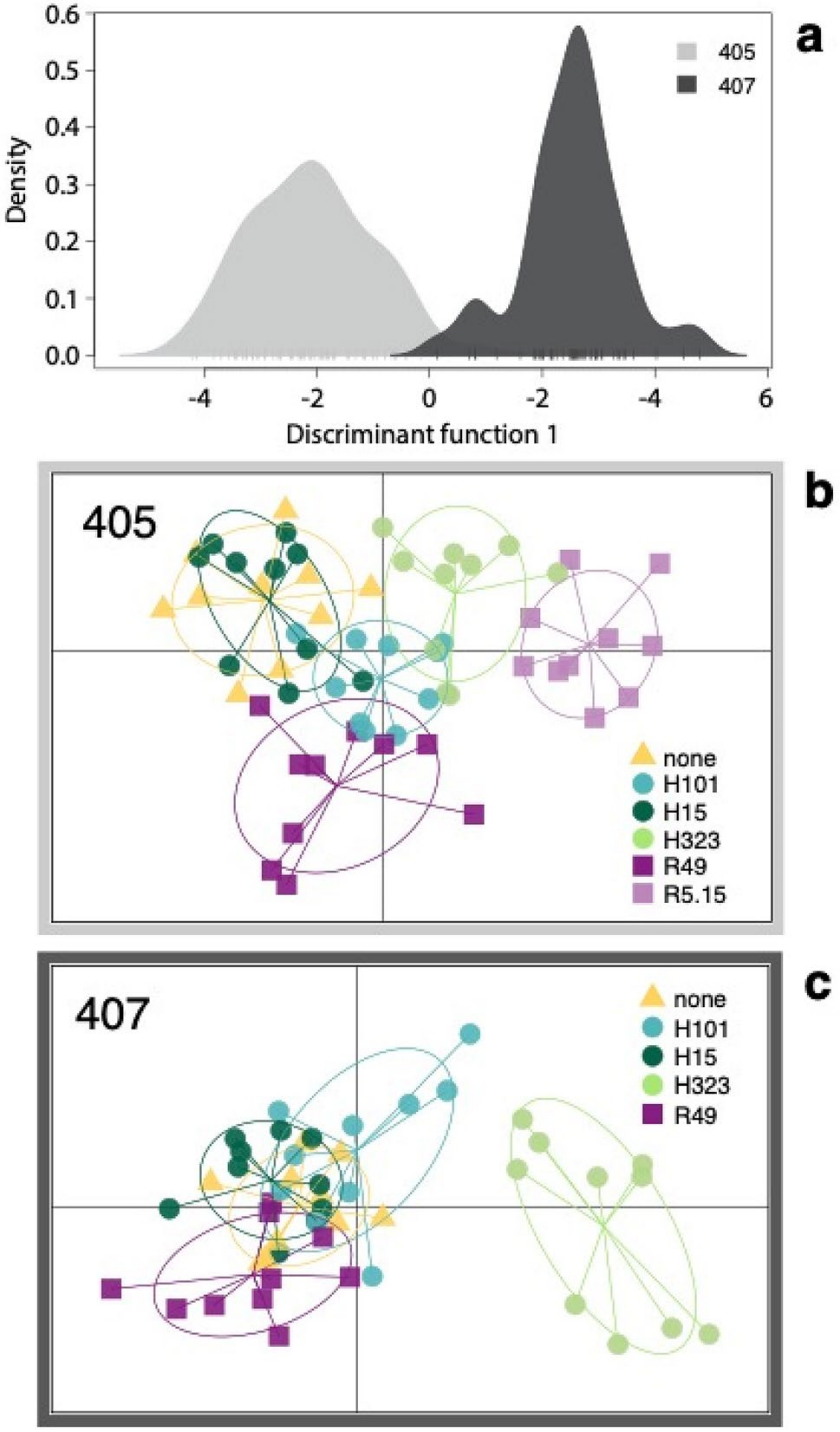
DAPC of aphid cuticular hydrocarbon profiles. (**a**) Shows the clear separation between aphid clones 405 and 407 (using 7 PCs). (**b**) Shows clone 405 without endosymbionts (yellow) and with different endosymbiont infections (using 16 PCs). (**c**) Shows clone 407 without (yellow) and with different endosymbiont infections (using 7PCs).

We identified 25 different compounds in the aphid CHC profile, including n-alkanes and monomethyl-, dimethyl-, and trimethyl-alkanes (supplementary material, table S1, figure S6 and S7). A DAPC on the clr-transformed proportions of all 25 compounds showed clear separation between the two aphid clones (Fig. 1a), which was supported by a highly significant clone effect in the PERMANOVA (pseudo-F = 74.00, R^2^ = 0.32, *P* = 0.001). There was also significant variation among endosymbiont infections (pseudo-F = 5.60, R^2^ = 0.12, *P* = 0.001), as well as a significant aphid clone x endosymbiont interaction (pseudo-F = 2.92, R^2^ = 0.05, *P* = 0.004), although the interaction explained less of the observed variation than clone or endosymbiont infection. We also found a significant block effect (pseudo-F = 3.39, R^2^ = 0.13, *P* = 0.001). Endosymbiont infections did not significantly change the total amount of CHCs per mg of aphid in either of the clones (supplementary material table S1, ANOVA; 405: F = 0.46, ndf = 5, ddf = 54, *P* = 0.81, 407: F = 1.08, ndf = 4, ddf = 45, *P* = 0.38). The observed chemical differences among clones and endosymbiont infections were based on varying ratios of compounds, as both aphid clones produced the same compounds (supplementary material, table S1).

### Changes in the CHC profile of clone 407

**Figure 2:**
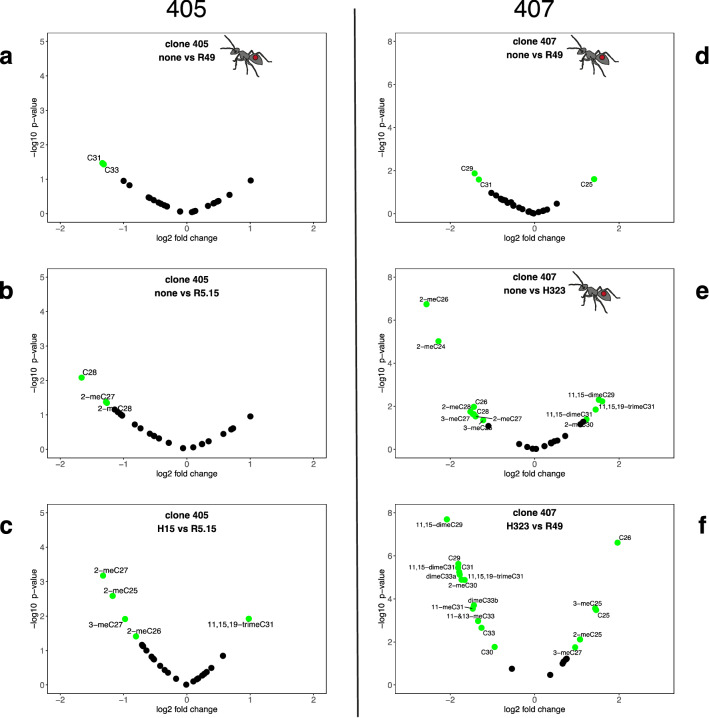
Volcano plots highlight the differences between uninfected and endosymbiont infected aphids (**a**, **b**,**d**,**e**) and between aphids with different endosymbiont infections (**c**,**f**). The left column shows comparisons within clone 405 and the right column within clone 407. Points highlighted in green represent compounds with *P* values < 0.05 and log-fold changes > 0.5. The combinations in plots (**a**),(**d**) and (**e**) were used in the behavioural ant experiments.

For clone 407, we found a large effect of endosymbiont infections on aphid CHC profiles (PERMANOVA: pseudo-F = 6.7, R^2^ = 0.35, *P* = 0.001) and only a weak blocking effect (block: pseudo-F = 1.60, R^2^ = 0.19, *P* = 0.05). Furthermore, we found a significant effect of endosymbiont species (all *Hamiltonella* and all *Regiella* strains pooled together) on the CHC profiles (PERMANOVA: pseudo-F = 2.52, R^2^ = 0.13, *P* = 0.04). A pairwise comparison showed that aphids infected with H323 or with R49 were significantly different from uninfected aphids (pairwise PERMANOVA, none:H323: pseudo-F = 9.09, R^2^ = 0.3, *P* = 0.001; none:R49: pseudo-F = 5.27, R^2^ = 0.18, *P* = 0.002) (Fig. 1c and supplementary material, table S2B). An infection with R49 increased the n-alkanes C29 and C31 while it decreased C25 (Fig. 2d). *H. defensa* H323, on the other hand, substantially increased the ratios of short monomethyl-branched compounds and the less abundant n-alkanes C26 and C28 while it decreased long, mainly di- and trimethyl-branched compounds (Fig. 2e).

Additionally, we found large differences between aphids that were infected with different endosymbionts. Aphids infected with H323 were significantly different from aphids infected with H15 (H323:H15: pseudo-F = 9.8, R^2^ = 0.36, *P* = 0.001, figure S3G), H101 (H323:H101: pseudo-F = 5.28, R^2^ = 0.24, *P* = 0.007, figure S3D), and R49 (H323:R49: pseudo-F = 17.11, R^2^ = 0.43, *P* = 0.001, Fig. 2f), while aphids infected with R49 were significantly different from aphids infected with H15 (R49:H15: pseudo-F = 3.36, R^2^ = 0.14, *P* = 0.014, figure S3F) and H101 (R49:H101: pseudo-F = 5.31, R^2^ = 0.19, *P* = 0.009, figure S3E) (overview in Fig. 1c, all pairwise comparison in supplementary material, table S2B and figure S3).

### Changes in the CHC profile of clone 405

For clone 405, we found an overall significant effect of endosymbiont infection on aphid CHC profiles (PERMANOVA: pseudo-F = 2.05, R^2^ = 0.13, *P* = 0.01) and a stronger blocking effect (pseudo-F = 2.45, R^2^ = 0.28, *P* = 0.001). Although we could not find a significant effect of endosymbiont species on the aphid CHC profiles (PERMANOVA: pseudo-F = 1.19, R^2^ = 0.04, *P* = 0.3), the CHC profiles from aphids infected with one of the two *R. insecticola s*trains (R49 or R5.15) were significantly different from CHCs of uninfected aphids (pairwise PERMANOVA, none:R49: pseudo-F = 2.85, R^2^ = 0.08, *P* = 0.042; none:R5.15: pseudo-F = 2.31, R^2^ = 0.08, *P* = 0.046)(Fig. 1b, supplementary material, table S2A). R49 increased the percentages of C31 and C33 (Fig. 2a) while R5.15 increased the percentages of monomethyl-branched compounds and C28, one of the less abundant linear alkanes (Fig. 2b).

We found more pronounced differences between aphids infected with different endosymbionts. H15 infected aphids had CHCs that were significantly different from H101 (H15:H101: pseudo-F = 3.94, R^2^ = 0.14, *P* = 0.017, figure S4F), H323 (H15:H323: pseudo-F = 2.43, R^2^ = 0.11, *P* = 0.023, figure S4H) and R5.15 (H15:R5.15: pseudo-F = 3.15, R^2^ = 0.13, *P* = 0.006, Fig. 2c) (overview in Fig. 1b, all pairwise comparison in supplementary material, table S2A, figure S4).

Since endosymbiont strains affect aphid CHCs in different ways, it is not surprising that, in both clones, we find the most extreme differences in CHC composition between aphids infected with different *R. insecticola* and *H. defensa* strains (Fig. 2c & f, supplementary material, figures S3 and S4).

### Ants can discriminate between CHC profiles of infected and uninfected aphids

**Figure 3:**
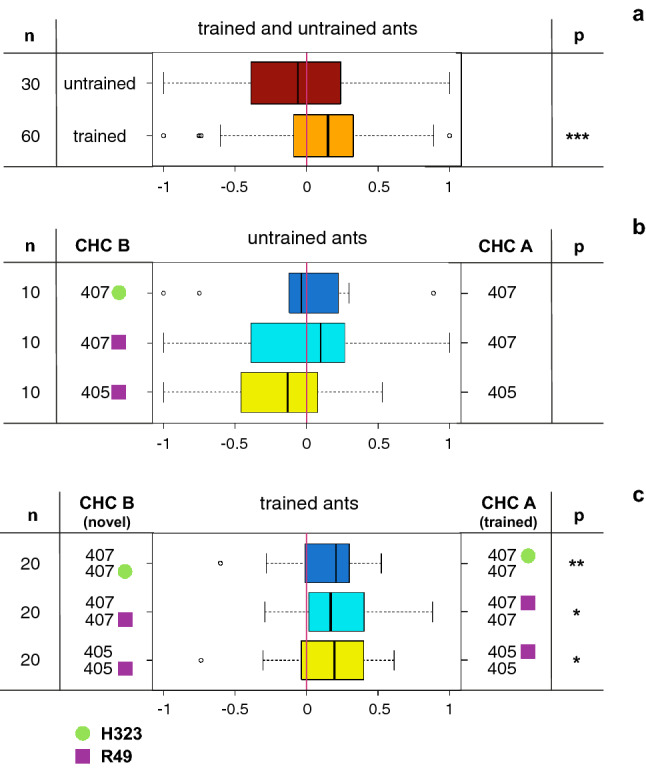
Preference indices (PI) of ant behavioral experiments. A PI of zero means no preference, a PI above or below zero a preference for CHC A (trained), or CHC B (novel), respectively. The number of replicates is indicated by n. Green dots represent an infection with *H. defensa* strain H323 and purple dots an infection with *R. insecticola* strain R49. (**a**) Shows a global plot of trained versus untrained ants. (**b**) Shows the absence of preferences of untrained ants. (**c**) Shows the preference of trained ants. Half of them were trained to the CHCs of uninfected aphids, the other half to CHCs of infected aphids. Asterisks show significant differences from zero (*P* value, * < 0.05, ** < 0.005, *** < 0.0005). Exact *P* values are mentioned in the text.

The preference index (PI) of trained ants was significantly larger than zero (Fig. 3a), indicating a clear effect of training on ant preferences for CHC A (t-test: t = 4.48, df = 59, *P* < 0.001). In contrast, untrained ants did not show a preference for either profile (Wilcoxon rank-sum test: W = 495, *P* = 0.48), nor did they exhibit an innate preference for any of the tested CHC profiles (Fig. 3b), as the PI in all combinations was not different from zero (t-tests: 405:405R49 t =  − 1.34, df = 9, *P* = 0.21; 407:407R49 t =  − 0.28, df = 9, *P* = 0.79; 407:407H323 t =  − 0.24, df = 9, *P* = 0.81). In contrast, trained ants invariably preferred the CHC profile to which they were trained (Fig. 3c). The PI values for trials with trained ants were significantly different from zero for all CHC combinations (t-test: 407:407H323 t = 3.5, df = 19, *P* = 0.001; 407:407R49 t = 2.3, df = 19, *P* = 0.015; 405:405R49 t = 2.0, df = 19, *P* = 0.028). It bears noting that the PIs for trials in which ants were trained to profiles of infected aphids were invariably higher than those in which ants were trained to the profiles of uninfected aphids (supplementary materials, figure S5). However, an analysis across all combinations showed that neither CHC combination nor whether ants were trained to endosymbiont infected or uninfected aphid lines had a significant effect on the PI of trained ants (factorial ANOVA: combination df = 2, MS = 0.02, F = 0.27, *P* = 0.764; infection df = 1, MS = 0.21, F = 2.48, *P* = 0.121). The interaction was also non-significant (combination x infection df = 2, MS = 0.04, F = 0.47, *P* = 0.636). This indicates that ants learned all CHC profiles equally well and that they were able to distinguish between CHC profiles with varying methyl-branched alkane ratios (none-H323) and also between profiles where only the linear alkane composition changed (none-R49).

## Discussion

Our results demonstrate that facultative endosymbiotic bacteria can change the composition of aphid CHCs in ways that are perceivable by tending ants. The observed effects on CHC profiles varied with the genetic background of the aphids as well as the endosymbiont strain present. Both *H. defensa* and *R. insecticola* strains had significant, and sometimes divergent, effects on the CHC profiles of their aphid hosts, although endosymbiont effects were smaller in *A. fabae* clone 405 than clone 407. In behavioural experiments, *L. niger* ants were able to learn to associate particular CHC profiles with rewards and discriminated between chemically divergent CHC profiles of endosymbiont infected and uninfected aphids of both clones. These results suggest that differences in CHC composition of endosymbiont infected and uninfected aphids might be a useful source of information for ants evaluating potential aphid mutualists.

Aphid clones 405 and 407 exhibited significantly different CHC profiles that were altered in clone-dependent ways by the presence of different endosymbionts. Clone-dependent effects of endosymbionts were also reported in a previous study showing that infection with different *H. defensa* strains imposed different life-history costs (reduced lifespan and reproduction) on aphid clones 405 and 407, perhaps due to resource competition between host and endosymbiont^20^. A blocking effect that mainly influenced samples from aphid clone 405 made it more difficult to find consistent patterns in the observed changes in this clone. This blocking effect (which was apparent even for samples from clone 405 reared together in blocks with clone 407) might be explained if clone 405 is more sensitive to slight differences in plant quality or environmental conditions. Despite this effect, we found that the two *R. insecticola* strains (R49 and R5.15) had the largest effects on CHC profiles in clone 405. For clone 407 the largest effects were caused by *R. insecticola* strain R49 and *H. defensa* strain H323.

The most pronounced feature of the chemical variation among our treatments was the observed reduction in long, dimethyl-, and trimethyl-branched compounds and an increase in short monomethyl-branched compounds in aphids of clone 407 infected with H323. As noted above, endosymbiont effects on aphid CHCs might be explained by competition for nutrients like lipids and amino acids between the endosymbionts and their hosts. CHC biosynthesis is closely linked to lipid metabolism and to the availability of some amino acids^29^. Insect CHCs are synthesized by elongating fatty acyl-CoAs to very long-chain fatty acids that are then converted to hydrocarbons^3,28^. A reduction in levels of fatty acyl-CoAs might thus explain the observed reduction of long-chain CHCs. Meanwhile, internally branched CHCs, like the dimethyl- and trimethyl-branched compounds mentioned above, result from the incorporation of a propionyl-CoA instead of an acetyl-CoA group^28^, while 2-methyl CHCs arise from the elongation of the carbon skeleton of valine (even numbers of C in the backbone) or isoleucine (odd numbers of C in the backbone)^29^. Effects of endosymbiotic bacteria on the availability of propionyl-CoA or the mentioned amino acids could thus explain the observed differences in methyl-branched compounds.

Our limited understanding of aphid CHC biosynthesis and of the nutritional requirements of different endosymbiont strains constrain our ability to provide a more detailed explanation of how endosymbionts may cause specific changes in aphid CHCs. However, there is evidence that endosymbionts likely affect the availability of CHC precursors. For example, competition for lipids between host and endosymbionts has been documented in *Drosophila* and mosquitos, where the proliferation of endosymbiotic bacteria is limited by lipid availability in the host haemolymph and fat-body^27,42,43^. In aphids, an overall reduction of amino acid concentration in *A. fabae* aphid honeydew was associated with infection by *H. defensa* and *R. insecticola*^10^. Specifically, the concentration of valine was reduced significantly by *H. defensa* and isoleucine was reduced by *H. defensa* and *R. insecticola,* albeit not significantly in the latter case. Since both amino acids are used in the biosynthesis of 2-methyl CHCs, this provides circumstantial evidence that facultative aphid endosymbionts influence the amino acid availability in their hosts and thus potentially the biosynthesis of methyl-branched CHCs.

Differences in the nutritional value of honeydew and differing CHC profiles between endosymbiont infected and uninfected aphids could, if detectable by ants, be a valuable informational source influencing the ant-aphid mutualism. Ants, for example, selectively prey on aphids that produce lower quality honeydew, and honeydew quality can be influenced by endosymbiont infections^10,38,39^. An important limitation of the current study is that we were not able to directly assess how potential effects of endosymbionts on honeydew quality influence ant preferences; however, the current results do demonstrate that ants are capable of detecting endosymbiont mediated changes in CHC profiles that are much smaller than the variation observed among aphid clones.

Furthermore, our results show that ants can perceive the differences in CHC profiles of endosymbiont-infected and uninfected aphids from both clones. The comparisons we used to train the ants showed the largest differences between infected and uninfected aphids and while R49 changed the ratios of linear alkanes, H323 changed mainly the methyl-branched compounds. Even the less pronounced differences affecting only linear alkanes that we observed in clone 405 were sufficient to enable ant learning. This is particularly notable given that linear alkanes are thought to play a less important role in communicative interactions than methyl-branched compounds^44^. Because of time constraints, we focussed on training ants to distinguish CHC profiles of infected and uninfected aphids only, but our chemical results showed that comparisons between aphids with different endosymbiont infections showed even larger chemical differences. It also bears noting that we consistently observed stronger ant preferences (higher PIs) in trials where ants had been trained to the CHC profiles of endosymbiont-infected aphids. While this pattern was not statistically significant, it at least raises the possibility that ants might more readily learn the profiles of endosymbiont infected aphids. Since the total amount of CHCs on endosymbiont infected and uninfected aphids was comparable, or even slightly lower in infected aphids, the asymmetric ant response was not based on quantitative but on compositional differences in CHCs. Similar asymmetries in ant learning and recognition of individual chemical compounds have been shown in other studies using different ant species (*Camponotus aethiops*^45^*; Linepithema humile*^46^). Individual ant learning has also been shown to influence collective foraging decisions in *L. niger* ants where even weak tendencies to learn the routes to high-quality food faster resulted in overall sensible colony decisions^47^.

Endosymbiont effects on CHCs might also have relevance for other aspects of the ant-aphid mutualism. For example, a previous study^48^ noted that ants seemed to selectively prey on *H. defensa* free aphids that were parasitized by parasitoid wasps. Parasitoids can have devastating effects in aphid colonies^49^ and removing infected individuals protects the whole colony. Harbouring *H. defensa* and some *R. insecticola* strains makes aphids highly resistant to parasitoid wasps^18,19^ so that there is no need for the ants to remove their mutualists when they are protected by endosymbionts*.* The mechanism by which ants distinguish between *H. defensa* infected and uninfected parasitized aphids has not been investigated yet but our results suggest that aphid CHC profiles are good candidates.

Endosymbiont-induced changes in aphid CHCs could also have implications for interactions with non-mutualists. For example, in the interactions discussed above, parasitoid wasps themselves might benefit by being able to detect the presence of endosymbionts that enhance aphid resistance. Indeed, there is evidence that the parasitoids *Aphidius ervi* and *Ephedrus plagiator* can distinguish aphids carrying *H. defensa*, albeit possibly via non-CHC cues^50,51^. Meanwhile, a recent study found no evidence for discrimination by *Lysiphlebus fabarum*, the main parasitoid of the *A. fabae* aphids studied here^52^. However, given the current finding that ants can detect endosymbiont-mediated variation in aphid CHCs and considerable evidence that endosymbionts also influence a wide range of ecologically relevant host traits^11,53^, CHC variation associated with the presence of different endosymbionts warrants further investigation.

In summary, the current findings provide the first evidence that aphid endosymbionts alter the CHC composition of their hosts. Specifically, we found that some *H. defensa* and *R. insecticola* strains significantly changed the proportions of short versus long CHCs and of methyl-branched alkanes while another *R. insecticola* strain affected the ratios of the linear alkanes only. Together with the finding that aphid-tending ants can perceive the differences between CHC profiles of endosymbiont infected and uninfected aphids, these findings provide new insight into the communicative functions of insect CHC profiles and suggest that they may play an important role in ant-aphid mutualism that goes beyond distinguishing myrmecophilous from non-myrmecophilous aphids and are possibly involved in other interspecific ecological interactions.

## Material and methods

### Aphid rearing experiment

We studied the effect of secondary endosymbiotic bacteria on the CHCs of two monoclonal lines of *Aphis fabae* (designated 405 and 407) that were either infected or uninfected with an endosymbiont. Both aphid clones were collected in Switzerland in 2006^54^ and maintained on *Vicia faba* beans (Fuego variety, seeds were obtained from Norddeutsche Pflanzenzucht, Malchow, Germany) in a climatized room under summer-like conditions (16 h light: 8 h dark, 18–20 °C). Aphid clones were free of any known secondary endosymbionts prior to artificial infection with one of three strains of *Hamiltonella defensa* (H15, H101, H323) or one of two strains of *Regiella insecticola* (R49, R5.15), via well-established microinjection techniques^19^. Donor aphids from H15, H101, H323, and R49 were European *A. fabae* while R5.15 was taken from an Australian *Myzus persicae* clone. Infection with strain R5.15 succeeded only in aphid clone 405. Artificial infections of the aphid lines were performed at least 2 years prior to the initiation of our experiments, and infection status was confirmed by regular testing over the intervening period. We reared ten replicate colonies of the two uninfected and nine infected *A. fabae* lines on *Vicia faba* plants, blocked in separate trays to account for tray or positioning effects, for three generations (to minimize maternal effects). For the chemical analysis we freeze-killed and pooled eight third-generation offspring from each replicate of the infected and uninfected lines as soon as they reached adulthood. All field collections and experiments with insects and plants comply with the ETH Zürich Guidelines for research integrity RSETHZ 414.

### Chemical analysis of aphid cuticular hydrocarbons (CHC)

Aphid samples were stored at − 80 °C until CHC extraction. We thawed and dried aphids for ~ 10 min before immersing them in 200 μL of hexane (three 5 min immersions), then collected the crude extract (600 μL) in a clean vial and applied it onto a 0.1 g SiOH column (silica gel 60, 230–400 mesh ASTM, particle size 0.04–0.063 mm, Fluka) to obtain only the nonpolar fractions. Next, we used 1 mL of hexane to elute the CHC from the column and dried the samples under a gentle flow of nitrogen to remove remaining volatile compounds. Samples were then resuspended in hexane and transferred into a low-volume glass insert where their volume was reduced to 24 μL of hexane, with 4 ng/μL of nonyl acetate as an internal standard. We analysed 2 μL of each sample on an Agilent GC–MS (Agilent 7890B/5977A GC-MSD (EI), Agilent Technologies AG) equipped with a DB-1 silica capillary column (30 m × 0.25 mm ID × 0.5 μm film thickness, Agilent Technologies AG). Helium was used as a carrier gas at a constant flow rate of 2 mL/min. We set the inlet temperature to 250 °C and the split/splitless injector to pulsed splitless mode. The electron impact mass spectra were measured at 70 eV. We heated the column with the following program: 60 °C for 2 min, 60–200 °C at a rate of 60 °C/min, 200–250 °C at a rate of 8 °C/min, 250–320 °C at a rate of 4 °C/min, and 320 °C for 10 min. Data for both MS and FID were collected simultaneously and analysed using Mass Hunter Software (Agilent technologies). We identified the linear n-alkanes by comparing their mass spectra and retention times to a C8-C40 alkane calibration standard (Supelco, USA). For identification of the methyl-branched alkanes we used Kovats’ retention indices^55,56^ and characteristic ions. We detected 25 different compounds belonging to the n-alkanes, monomethyl-, dimethyl-, and trimethyl-alkanes.

### Ant behavioural experiments

For behavioural experiments, we used *Lasius niger* ants from five queenright colonies collected in Switzerland in 2014 and 2015 and maintained in open plastic boxes (32.5 × 17.6 × 15 cm) with test tube nests (1.5 × 15 cm, half filled with water and plugged with cotton wool). A stripe of Teflon™ PTFE DISP30 Fluoropolymer Dispersion (Chemours) made the boxes escape-proof. Colonies were maintained under a 16 h light, 8 h dark cycle at 22–23 °C and given 10% honey water ad libitum and mealworms (*Tenebrio molitor*) twice weekly. Ants were starved for 3 days before behavioural experiments, and we separated forager ants that were to be used for each experiment from the colonies and marked them with water-based paint (POSCA colouring pens).

To test whether ants are able to distinguish between symbiont-infected and uninfected aphids, we focused on three combinations that showed strong chemical differences (405–405R49, 407–407R49, 407–407H323). We trained ants to associate a reward to aphid CHC profiles, before testing the responses of trained ants (vs untrained controls) to different CHC profiles in the absence of rewards (assay adapted from^45^). Training and test trials took place in plastic petri dishes (10 cm diameter, 1.5 cm high) with Teflon-coated walls. Filter paper disks covering the bottom of the dish were replaced after every trial. For training, we placed two round microscope cover glasses (18 mm) in the petri dish. On the edge of one of the cover glasses, we applied CHCs corresponding to 20 adult aphids (dissolved in 10 μl of hexane, extraction method as described above). Well after the hexane evaporated, a reward (a drop of 10% honey water) was placed in the centre, so that ants walked over the CHCs to reach it. The second cover glass was clean (supplementary material, figure S1). We used new glasses for every training trial and randomized their positions. Individual, marked ants were released in the petri dish. After they found the honey reward and filled their gaster, we moved them back to their colony and let them perform trophallaxis with their nest mates, which took ~ 1 min if they directly passed on the reward to other foragers, or up to 30 min when they entered the nest. When the ant started foraging again, it was moved back to the petri dish for the next training trial. Each ant received six consecutive training trials on the same day. Half the ants were trained to the CHC profiles of uninfected aphids (10 ants to 405, 20 ants to 407) and the other half to those of endosymbiont-infected aphids (10 ants to 405R49, 407R49, and 407H323 each).

Immediately following the six training trials, each ant performed two test trials to assess its learned preference. In test trials, one cover glass carried the CHC profile they were trained to (CHC A) and the other one the novel CHC profile of the corresponding infected or uninfected aphid line (CHC B). Neither of the glasses provided a reward (supplementary material, figure S1). Again, we used new glasses for each test and randomized their positions. Using NOLDUS observer software, we recorded the location of the ant (cover glass with CHC A, cover glass with CHC B, or empty space around) for 3 min. Afterwards, ants received a honey reward in the centre of the cover glass with CHC A before being transferred back to the colony to perform trophallaxis. Ants performed a second test trial (to exclude a bias for the location of the reward in the last training trial) after which they were kept separate from the colony until the end of the experiment. Control ants were taken from the same colonies, marked with paint, but received no training. We introduced them once into petri dishes with a pair of cover glasses that were coated with 405 and 405R49, 407 and 407R49, or 407 and 407H323 but neither provided a honey reward. We recorded their location for 3 min to find out whether they had an innate preference for one of the profiles. At the end of the experimental days, we returned them to their colonies.

### Statistical analysis

All the statistical analyses were performed in R version 4.0.3^57^.

#### Analysis of aphid cuticular hydrocarbons

We first converted the area of each of the 25 detected peaks in the chromatograms to their proportional contribution to the total peak area of every sample. Data were then visualized using discriminant analysis of principal components (DAPC)^58^. To get a strongly discriminating and stable solution without over-fitting the data, we used the optim.a.score function (adegenet 2.0.0 package^59^) to determine the number of used principal components.

Next, we centre-log-ratio (clr) transformed the proportions before using all 25 compounds as response variables for a permutational analysis of variance (PERMANOVA)^60^ based on Euclidean distances. In a global analysis, we used aphid clone (405 or 407) and endosymbiont infection (uninfected, H15, H101, H323, R49 and R5.15), as well as their interaction, and block as explanatory variables. The multivariate sample dispersion of the aphid clones was homogenous, while the dispersion in the groups with different endosymbiont infections was heterogenous. However, since the smaller group (R5.15) also had a smaller dispersion the test was rather too conservative^61^. Because the aphid clones proved to be significantly different from each other, we performed a PERMANOVA followed by a post hoc test (pairwise.adonis2 function^62^) for each aphid clone separately. We corrected the *P* values using the Benjamini & Hochberg false discovery rate method. Within the two aphid clones, groups infected with different endosymbionts had a heterogeneous dispersions, but for balanced designs this does not affect the result of the PERMANOVA analysis^61^. To look at differences in compound ratios between the infected and uninfected lines, we used the untransformed data and the eBayes function (limma package^63^) to calculate *P* values and log-fold changes that we visualized using volcano plots.

#### Analysis of ant behaviour data

From the time the ants spent on CHC profile A (trained) and CHC profile B (novel) we calculated a preference index (PI) for CHC A using the following formula: PI = ((time CHC A − time CHC B) / (time CHC A + time CHC B)). A PI of zero indicates no preference, a positive PI a preference for CHC A and a negative PI a preference for CHC B. To detect possible differences between the first and second ant tests, we fitted a linear mixed effects model (lmerTest) with PI as response variable, test 1 or 2 as fixed effect, and ant individual as random effect, followed by a type three ANOVA with Satterthwaite’s degrees of freedom method. Since we could not find a significant difference between test 1 and 2 (ndf = 1, ddf = 59, MS = 0.02, F = 0.196, *P* = 0.659), we calculated the mean of both tests for every individual ant and continued using these values. To find out whether the ants were able to discriminate between CHC profile A and the novel CHC profile B, we compared the PI of all trained ants, and all different comparisons, to zero using one-sided t-tests. Because the PI of the untrained ants were not normally distributed, we used two-sided Wilcoxon rank-sum tests to test for significant differences from zero. To investigate whether the PI of ants trained to CHCs of uninfected aphids were significantly different from those of ants trained to CHCs of infected aphids, we ran a factorial ANOVA with PI as response variable and treatment combination, infection status, and their interaction as explanatory variables.

## Supplementary Information

Supplementary Information.

## Data Availability

The data that support the findings of this study are openly available in the ETH Zürich Research Collection at http://doi.org/10.3929/ethz-b-000477293 .

## References

[1] Gibbs, A. G. & Rajpurohit, S. Cuticular lipids and water balance. in *Insect hydrocarbons: biology, biochemistry, and chemical ecology* 100–120 (Cambridge University Press Cambridge, UK, 2010). 10.1017/CBO9780511711909.007

[2] Pedrini N, Ortiz-Urquiza A, Zhang S, Keyhani NO (2013). Targeting of insect epicuticular lipids by the entomopathogenic fungus Beauveria bassiana: hydrocarbon oxidation within the context of a host-pathogen interaction. Front. Microbiol..

[3] Howard RW, Blomquist GJ (2005). Ecological, behavioral, and biochemical aspects of insect hydrocarbons. Annu. Rev. Entomol..

[4] Lang C, Menzel F (2011). Lasius niger ants discriminate aphids based on their cuticular hydrocarbons. Anim. Behav..

[5] Sakata I, Hayashi M, Nakamuta K (2017). Tetramorium tsushimae ants use methyl branched hydrocarbons of aphids for partner recognition. J. Chem. Ecol..

[6] Salazar A (2015). Aggressive mimicry coexists with mutualism in an aphid. Proc. Natl. Acad. Sci..

[7] Endo S, Itino T (2012). The aphid-tending ant Lasius fuji exhibits reduced aggression toward aphids marked with ant cuticular hydrocarbons. Popul. Ecol..

[8] Endo S, Itino T (2013). Myrmecophilous aphids produce cuticular hydrocarbons that resemble those of their tending ants. Popul. Ecol..

[9] Stadler B, Dixon AFG (2005). Ecology and evolution of aphid-ant interactions. Annu. Rev. Ecol. Evol. Syst..

[10] Schillewaert S (2017). The influence of facultative endosymbionts on honeydew carbohydrate and amino acid composition of the black bean aphid Aphis fabae. Physiol. Entomol..

[11] Oliver KM, Degnan PH, Burke GR, Moran NA (2010). Facultative symbionts in aphids and the horizontal transfer of ecologically important traits. Annu. Rev. Entomol..

[12] Douglas AE (1998). Nutritional interactions in insect-microbial symbioses: aphids and their symbiotic bacteria Buchnera. Annu. Rev. Entomol..

[13] Montllor CB, Maxmen A, Purcell AH (2002). Facultative bacterial endosymbionts benefit pea aphids Acyrthosiphon pisum under heat stress. Ecol. Entomol..

[14] Russell JA, Moran NA (2005). Costs and benefits of symbiont infection in aphids: variation among symbionts and across temperatures. Proc. R. Soc. B Biol. Sci..

[15] Wagner SM (2015). Facultative endosymbionts mediate dietary breadth in a polyphagous herbivore. Funct. Ecol..

[16] Scarborough CL, Ferrari J, Godfray HCJ (2005). Aphid protected from pathogen by endosymbiont. Science.

[17] Łukasik P, van Asch M, Guo H, Ferrari J, Godfray HCJ (2013). Unrelated facultative endosymbionts protect aphids against a fungal pathogen. Ecol. Lett..

[18] Oliver KM, Russell JA, Moran NA, Hunter MS (2003). Facultative bacterial symbionts in aphids confer resistance to parasitic wasps. Proc. Natl. Acad. Sci..

[19] Vorburger C, Gehrer L, Rodriguez P (2010). A strain of the bacterial symbiont Regiella insecticola protects aphids against parasitoids. Biol. Lett..

[20] Vorburger C, Gouskov A (2011). Only helpful when required: a longevity cost of harbouring defensive symbionts. J. Evol. Biol..

[21] Vorburger C, Ganesanandamoorthy P, Kwiatkowski M (2013). Comparing constitutive and induced costs of symbiont-conferred resistance to parasitoids in aphids. Ecol. Evol..

[22] Gwynn DM, Callaghan A, Gorham J, Walters KFA, Fellowes MDE (2005). Resistance is costly: trade-offs between immunity, fecundity and survival in the pea aphid. Proc. R. Soc. B Biol. Sci..

[23] Oliver KM, Campos J, Moran NA, Hunter MS (2008). Population dynamics of defensive symbionts in aphids. Proc. R. Soc. B Biol. Sci..

[24] Wernegreen JJ (2002). Genome evolution in bacterial endosymbionts of insects. Nat. Rev. Genet..

[25] Degnan PH, Yu Y, Sisneros N, Wing RA, Moran NA (2009). Hamiltonella defensa, genome evolution of protective bacterial endosymbiont from pathogenic ancestors. Proc. Natl. Acad. Sci..

[26] Ankrah NYD, Luan J, Douglas AE (2017). Cooperative metabolism in a three-partner insect-bacterial symbiosis revealed by metabolic modeling. J. Bacteriol..

[27] Herren JK (2014). Insect endosymbiont proliferation is limited by lipid availability. Elife.

[28] Hamilton RJ (1995). Waxes: Chemistry, Molecular Biology and Functions.

[29] Blailock TT, Blomquist GJ, Jackson LL (1976). Biosynthesis of 2-methylalkanes in the crickets: Nemobiusfasciatus and Grylluspennsylvanicus. Biochem. Biophys. Res. Commun..

[30] Engl T (2018). Effect of antibiotic treatment and gamma-irradiation on cuticular hydrocarbon profiles and mate choice in tsetse flies (Glossina m. morsitans). BMC Microbiol..

[31] Engl T (2018). Ancient symbiosis confers desiccation resistance to stored grain pest beetles. Mol. Ecol..

[32] Schneider DI (2019). Symbiont-driven male mating success in the Neotropical Drosophila paulistorum superspecies. Behav. Genet..

[33] de Souza DJ, Devers S, Lenoir A (2011). Blochmannia endosymbionts and their host, the ant Camponotus fellah: cuticular hydrocarbons and melanization. C. R. Biol..

[34] Richard F-J (2017). Symbiotic bacteria influence the odor and mating preference of their hosts. Front. Ecol. Evol..

[35] Fischer MK, Shingleton AW (2001). Host plant and ants influence the honeydew sugar composition of aphids. Funct. Ecol..

[36] Yao I, Akimoto S (2001). Ant attendance changes the sugar composition of the honeydew of the drepanosiphid aphid Tuberculatus quercicola. Oecologia.

[37] Yao I, Akimoto S (2002). Flexibility in the composition and concentration of amino acids in honeydew of the drepanosiphid aphid Tuberculatus quercicola. Ecol. Entomol..

[38] Offenberg J (2001). Balancing between mutualism and exploitation: the symbiotic interaction between Lasius ants and aphids. Behav. Ecol. Sociobiol..

[39] Stadler B, Dixon AFG (1999). Ant attendance in aphids: why different degrees of myrmecophily?. Ecol. Entomol..

[40] Vantaux A, Van den Ende W, Billen J, Wenseleers T (2011). Large interclone differences in melezitose secretion in the facultatively ant-tended black bean aphid Aphis fabae. J. Insect. Physiol..

[41] Moran NA, Russell JA, Koga R, Fukatsu T (2005). Evolutionary relationships of three new species of Enterobacteriaceae living as symbionts of aphids and other insects. Appl. Environ. Microbiol..

[42] Molloy JC, Sommer U, Viant MR, Sinkins SP (2016). Wolbachia modulates lipid metabolism in Aedes albopictus mosquito cells. Appl. Environ. Microbiol..

[43] Paredes JC, Herren JK, Schüpfer F, Lemaitre B (2016). The role of lipid competition for endosymbiont-mediated protection against parasitoid wasps in Drosophila. MBio.

[44] Chung H, Carroll SB (2015). Wax, sex and the origin of species: dual roles of insect cuticular hydrocarbons in adaptation and mating. BioEssays.

[45] Bos N (2012). Learning and perceptual similarity among cuticular hydrocarbons in ants. J. Insect Physiol..

[46] van Wilgenburg E (2012). Learning and discrimination of cuticular hydrocarbons in a social insect. Biol. Lett..

[47] Oberhauser FB, Koch A, Czaczkes TJ (2018). Small differences in learning speed for different food qualities can drive efficient collective foraging in ant colonies. Behav. Ecol. Sociobiol..

[48] Erickson DM, Wood EA, Oliver KM, Billick I, Abbot P (2012). The effect of ants on the population dynamics of a protective symbiont of aphids, Hamiltonella defensa. Ann. Entomol. Soc. Am..

[49] Schmidt MH (2003). Relative importance of predators and parasitoids for cereal aphid control. Proc. R. Soc. Lond. Ser. B. Biol. Sci..

[50] Łukasik P, Dawid MA, Ferrari J, Godfray HCJ (2013). The diversity and fitness effects of infection with facultative endosymbionts in the grain aphid, Sitobion avenae. Oecologia.

[51] Oliver KM (2012). Parasitic wasp responses to symbiont-based defense in aphids. BMC Biol..

[52] Dennis AB, Patel V, Oliver KM, Vorburger C (2017). Parasitoid gene expression changes after adaptation to symbiont-protected hosts. Evolution.

[53] Guo J (2017). Nine facultative endosymbionts in aphids, a review. J. Asia. Pac. Entomol..

[54] Vorburger C, Sandrock C, Gouskov A, Castañeda LE, Ferrari J (2009). Genotypic variation and the role of defensive endosymbionts in an all-parthenogenetic host–parasitoid interaction. Evol. Int. J. Org. Evol..

[55] Carlson DA, Bernier UR, Sutton BD (1998). Elution patterns from capillary GC for methyl-branched alkanes. J. Chem. Ecol..

[56] Katritzky AR, Chen K, Maran U, Carlson DA (2000). QSPR correlation and predictions of GC retention indexes for methyl-branched hydrocarbons produced by insects. Anal. Chem..

[57] R Core Team. R: A Language and Environment for Statistical Computing. (2019).

[58] Jombart T, Devillard S, Balloux F (2010). Discriminant analysis of principal components: a new method for the analysis of genetically structured populations. BMC Genet..

[59] Jombart T (2008). adegenet: a R package for the multivariate analysis of genetic markers. Bioinformatics.

[60] Anderson MJ (2014). Permutational multivariate analysis of variance (PERMANOVA). Wiley Statsref. Stat. Ref..

[61] Anderson MJ, Walsh DCI (2013). PERMANOVA, ANOSIM, and the Mantel test in the face of heterogeneous dispersions: what null hypothesis are you testing?. Ecol. Monogr..

[62] Arbizu, P. M. pairwiseAdonis: Pairwise Multilevel Comparison using Adonis (2017).

[63] Ritchie ME (2015). limma powers differential expression analyses for RNA-sequencing and microarray studies. Nucleic Acids Res..

