# Supplementary material for: Aphids harbouring different endosymbionts exhibit differences in cuticular hydrocarbon profiles that can be recognized by ant mutualists
Source: Sci Rep. 2021 Oct 1;11:19559. doi: 10.1038/s41598-021-98098-2 (PMC8486828; doi:10.1038/s41598-021-98098-2)
Supplement: Supplementary file 1 — Supplementary Information. [file 41598_2021_98098_MOESM1_ESM.pdf]

Supplementary Material:

Aphids harbouring different endosymbionts exhibit differences in cuticular hydrocarbon profiles that can be recognized by ant mutualists

Authors: Corinne Hertaeg[1,2], Marion Risse[1], Christoph Vorburger[1,2], Consuelo M. De Moraes[1], Mark C. Mescher[1*]

[1] Department of Environmental Systems Science, ETH Zürich, Zürich, Switzerland

[2] Department of Aquatic Ecology, Eawag, Swiss Federal Institute of Aquatic Science and Technology, Dübendorf, Switzerland

***Corresponding author:** Mark C. Mescher[1] (mescher@usys.ethz.ch)

Table S1. Average relative percentages (± standard deviation) of all compounds in the CHC profiles of the infected and uninfected aphid lines of clone 405 and 407. The compounds are sorted by retention time from lowest to highest. The last row of the table shows the total absolute amount of CHC [ng/µL/mg of aphid] that was determined by comparison with an internal standard.

**Compound**	**Aphid line**										
	**405**						**407**				
	**none**	**H101**	**H15**	**H323**	**R49**	**R5.15**	**none**	**H101**	**H15**	**H323**	**R49**
2-Me C24	1.83 ± 0.44	2.20 ± 0.83	1.49 ± 0.33	1.68 ± 0.75	1.51 ± 0.39	1.71 ± 0.70	2.73 ± 0.24	3.61 ± 1.33	2.62 ± 0.34	5.27 ± 0.84	2.08 ± 0.54
C25	19.80 ± 4.29	22.05 ± 6.85	18.33 ± 2.87	20.94 ± 4.61	17.94 ± 5.63	16.87 ± 2.94	26.98 ± 4.20	25.07 ± 5.11	25.59 ± 3.88	28.62 ± 4.90	21.59 ± 3.70
2-Me C25	1.22 ± 0.20	1.49 ± 0.57	1.04 ± 0.11	1.38 ± 0.31	1.01 ± 0.18	1.48 ± 0.39	2.33 ± 0.51	2.59 ± 0.66	2.18 ± 0.20	2.79 ± 0.57	2.24 ± 0.34
3-Me C25	1.37 ± 0.31	1.46 ± 0.48	1.10 ± 0.24	1.36 ± 0.41	1.05 ± 0.28	1.35 ± 0.52	1.60 ± 0.31	2.20 ± 1.10	1.61 ± 0.20	2.27 ± 0.65	1.27 ± 0.32
C26	1.30 ± 0.19	1.56 ± 0.39	1.31 ± 0.16	1.48 ± 0.17	1.35 ± 0.23	1.39 ± 0.23	1.67 ± 0.23	1.58 ± 0.18	1.64 ± 0.21	2.06 ± 0.26	1.50 ± 0.19
2-Me C26	1.99 ± 0.23	2.08 ± 0.26	1.89 ± 0.33	2.03 ± 0.18	1.95 ± 0.24	2.12 ± 0.44	2.41 ± 0.25	2.75 ± 0.28	2.34 ± 0.17	3.80 ± 0.47	2.21 ± 0.29
C27	12.40 ± 1.32	12.93 ± 1.65	12.42 ± 0.93	13.17 ± 1.24	12.37 ± 1.34	12.10 ± 1.37	14.25 ± 1.43	12.96 ± 0.93	14.34 ± 1.00	14.65 ± 1.17	14.19 ± 1.29
2-Me C27	0.61 ± 0.24	0.65 ± 0.22	0.53 ± 0.11	0.77 ± 0.26	0.68 ± 0.29	0.89 ± 0.36	0.77 ± 0.15	0.96 ± 0.76	0.86 ± 0.17	1.96 ± 2.15	1.12 ± 0.60
3-Me C27	0.75 ± 0.12	0.77 ± 0.13	0.74 ± 0.12	0.71 ± 0.07	0.68 ± 0.17	0.89 ± 0.21	0.70 ± 0.10	0.74 ± 0.11	0.75 ± 0.10	0.93 ± 0.39	0.73 ± 0.13
C28	0.83 ± 0.12	0.85 ± 0.13	0.93 ± 0.18	0.95 ± 0.19	0.91 ± 0.15	1.02 ± 0.16	0.78 ± 0.22	0.78 ± 0.11	0.85 ± 0.13	1.23 ± 0.65	0.98 ± 0.21
2-Me C28	0.83 ± 0.13	0.79 ± 0.18	0.87 ± 0.11	0.88 ± 0.15	0.92 ± 0.11	0.97 ± 0.15	0.71 ± 0.11	0.73 ± 0.13	0.70 ± 0.08	0.88 ± 0.22	0.77 ± 0.10
C29	10.93 ± 1.27	10.79 ± 1.78	12.39 ± 0.96	12.12 ± 1.38	10.98 ± 1.50	12.13 ± 2.38	7.53 ± 1.05	7.46 ± 1.15	8.43 ± 0.86	6.66 ± 1.16	9.21 ± 1.44
11-Me C29	3.92 ± 1.42	3.70 ± 0.94	3.66 ± 1.28	3.63 ± 1.30	3.46 ± 1.11	4.42 ± 2.00	4.32 ± 1.65	4.09 ± 1.42	3.73 ± 0.98	5.01 ± 2.56	3.93 ± 1.13
11,15-Dime C29	5.92 ± 0.95	5.47 ± 1.52	5.99 ± 1.10	5.06 ± 0.87	6.46 ± 1.62	5.77 ± 1.11	5.15 ± 0.92	5.40 ± 1.05	5.61 ± 0.91	3.24 ± 0.73	5.96 ± 0.97
Dime C29	0.75 ± 0.09	0.71 ± 0.12	0.78 ± 0.17	0.71 ± 0.16	0.68 ± 0.13	0.85 ± 0.24	0.68 ± 0.09	0.72 ± 0.09	0.74 ± 0.09	0.62 ± 0.14	0.68 ± 0.06
C30	0.51 ± 0.06	0.48 ± 0.04	0.54 ± 0.05	0.53 ± 0.07	0.52 ± 0.06	0.57 ± 0.06	0.42 ± 0.05	0.39 ± 0.06	0.40 ± 0.05	0.42 ± 0.08	0.48 ± 0.07
2-Me C30	1.36 ± 0.28	1.19 ± 0.34	1.38 ± 0.21	1.17 ± 0.23	1.40 ± 0.29	1.27 ± 0.17	1.17 ± 0.23	1.17 ± 0.28	1.20 ± 0.21	0.85 ± 0.26	1.34 ± 0.22
C31	3.32 ± 0.55	3.27 ± 0.70	3.75 ± 0.51	3.70 ± 0.48	3.86 ± 0.36	3.68 ± 0.84	1.72 ± 0.45	1.73 ± 0.55	1.89 ± 0.31	1.38 ± 0.35	2.32 ± 0.50
11-Me C31	5.00 ± 0.80	4.64 ± 0.53	4.84 ± 0.46	4.56 ± 0.52	5.48 ± 1.06	5.36 ± 0.59	3.89 ± 0.64	4.03 ± 0.65	3.93 ± 0.46	3.37 ± 0.68	4.36 ± 0.62
11,15-Dime C31	14.79 ± 2.79	13.48 ± 4.57	15.33 ± 2.17	13.43 ± 2.74	15.43 ± 3.28	14.38 ± 2.05	12.58 ± 2.73	13.03 ± 2.91	12.60 ± 1.97	8.51 ± 3.04	14.13 ± 1.71
11,15,19-Trime C31	1.65 ± 0.25	1.42 ± 0.40	1.74 ± 0.24	1.41 ± 0.35	1.61 ± 0.37	1.42 ± 0.26	1.28 ± 0.16	1.33 ± 0.31	1.37 ± 0.24	0.84 ± 0.27	1.35 ± 0.19
C33	0.81 ± 0.29	0.87 ± 0.31	0.93 ± 0.25	0.93 ± 0.25	1.05 ± 0.20	0.97 ± 0.30	0.49 ± 0.18	0.52 ± 0.28	0.53 ± 0.15	0.40 ± 0.19	0.69 ± 0.25
11- and 13-Me C33	3.03 ± 0.82	2.79 ± 0.58	2.92 ± 0.31	2.80 ± 0.49	3.25 ± 0.77	3.34 ± 0.38	2.09 ± 0.45	2.24 ± 0.55	2.10 ± 0.39	1.75 ± 0.55	2.50 ± 0.65
Dime C33 a	3.77 ± 0.97	3.23 ± 1.31	3.86 ± 0.52	3.49 ± 0.70	4.10 ± 1.00	3.87 ± 0.60	2.65 ± 0.64	2.82 ± 0.82	2.79 ± 0.55	1.70 ± 0.93	3.26 ± 0.70

Dime C33 b	1.32 ± 0.30	1.15 ± 0.31	1.35 ± 0.15	1.08 ± 0.29	1.36 ± 0.39	1.18 ± 0.24	1.06 ± 0.17	1.11 ± 0.28	1.19 ± 0.23	0.77 ± 0.12	1.14 ± 0.23
**TOTAL CHC [ng/µL] per mg of aphid**	**3.04 ± 0.86**	**2.95 ± 1.11**	**2.92 ± 0.81**	**2.59 ± 0.48**	**2.59 ± 0.95**	**2.90 ± 1.10**	**3.26 ± 1.00**	**3.22 ± 1.14**	**3.28 ± 1.06**	**2.46 ± 1.06**	**2.95 ± 1.01**

Table S2. Differences between uninfected aphids and aphids infected with different endosymbiont strains of (A.) clone 405 and (B.) clone 407. We performed a posthoc test of the PERMANOVA results and corrected the p-values with the Benjamini-Hochberg method. Asterisks (,**,***) indicate significant results <0.05, <0.005 and <0.0005, respectively. Dots (.) mark marginally significant results.*

A. Clone 405

**comparison**	**factor**	**pseudo F**	**R2**	**adjusted p-value**	
R5.15 : R49	endosymbiont	2.23	0.1	0.059	.
	block	1.34	0.52	0.169	
	residuals		0.39		
R5.15 : H101	endosymbiont	1.83	0.08	0.142	
	block	1.41	0.54	0.203	
	residuals		0.38		
R5.15 : H323	endosymbiont	0.73	0.04	0.626	
	block	0.83	0.43	0.791	
	residuals		0.52		
R5.15 : none	endosymbiont	2.31	0.08	0.046	*
	block	1.97	0.61	0.009	**
	residuals		0.31		
R5.15 : H15	endosymbiont	3.15	0.13	0.006	**
	block	1.34	0.5	0.131	
	residuals		0.37		
R49 : H101	endosymbiont	2.71	0.13	0.062	.
	block	0.99	0.43	0.466	
	residuals		0.44		
R49 : H323	endosymbiont	2.2	0.09	0.063	.
	block	1.51	0.55	0.07	.

B. Clone 407

**comparison**	**factor**	**pseudo F**	**R2**	**adjusted p-value**	
H323 : R49	endosymbiont	17.11	0.43	0.001	***
	block	1.53	0.34	0.154	
	residuals		0.23		
H323 : H15	endosymbiont	9.8	0.36	0.001	***
	block	0.93	0.31	0.561	
	residuals		0.33		
H323 : H101	endosymbiont	5.28	0.24	0.007	**
	block	0.87	0.35	0.637	
	residuals		0.41		
H323 : none	endosymbiont	9.09	0.3	0.001	***
	block	1.31	0.39	0.251	
	residuals		0.3		
R49 : H15	endosymbiont	3.36	0.14	0.014	*
	block	1.21	0.49	0.239	
	residuals		0.39		
R49 : H101	endosymbiont	5.31	0.19	0.009	**
	block	1.46	0.48	0.155	
	residuals		0.33		
R49 : none	endosymbiont	5.27	0.18	0.002	**
	block	1.73	0.52	0.04	*

	residuals		0.36		
R49 : none	endosymbiont	2.85	0.08	0.042	*
	block	2.55	0.66	0.004	**
	residuals		0.26		
R49 : H15	endosymbiont	1.36	0.04	0.191	
	block	2.27	0.66	0.002	**
	residuals		0.29		
H101 : H323	endosymbiont	1.06	0.05	0.343	
	block	1.22	0.52	0.303	
	residuals		0.43		
H101 : none	endosymbiont	1.45	0.05	0.235	
	block	2.01	0.63	0.059	.
	residuals		0.32		
H101 : H15	endosymbiont	3.94	0.14	0.017	*
	block	1.73	0.55	0.104	
	residuals		0.32		
H323 : none	endosymbiont	1.78	0.07	0.091	.
	block	1.46	0.55	0.082	.
	residuals		0.38		
H323 : H15	endosymbiont	2.43	0.11	0.023	*
	block	1.24	0.49	0.169	
	residuals		0.4		
none : H15	endosymbiont	2.36	0.07	0.062	.
	block	2.7	0.68	0.002	**
	residuals		0.25		

	residuals		0.3		
H15 : H101	endosymbiont	1.35	0.06	0.256	
	block	1.29	0.53	0.224	
	residuals		0.41		
H15 : none	endosymbiont	0.9	0.04	0.479	
	block	1.74	0.61	0.039	*
	residuals		0.35		
H101 : none	endosymbiont	0.74	0.04	0.566	
	block	1.22	0.53	0.257	
	residuals		0.43		

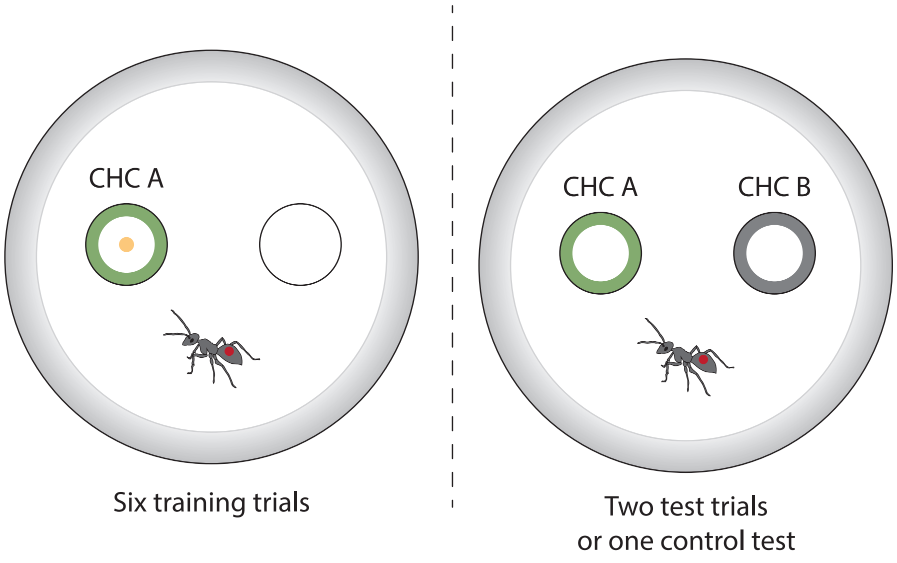

Figure S1 Experimental setup of the training trials on the left and the test trials on the right. The yellow circle depicts the honey water reward. CHC A refers to the CHC profile the ants were trained to. This could either be a profile from an uninfected or infected aphid. CHC B is the profile of the corresponding infected or uninfected aphid line which was novel for the ants. Control ants only experienced the setup on the right without ever getting training trials or a reward.

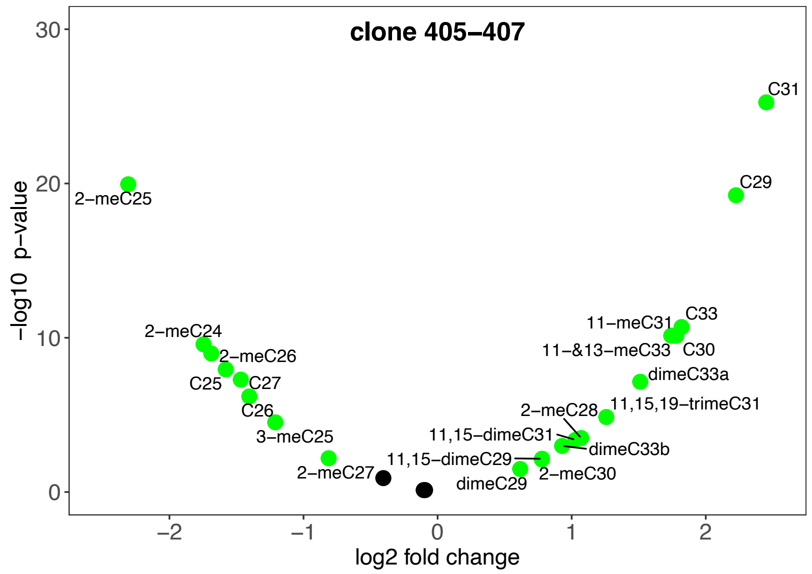

Figure S2 Volcano plot that highlights the differences between clones 405 and 407. Points highlighted in green represent compounds with p-values <0.05 and log-fold changes >0.5.

407

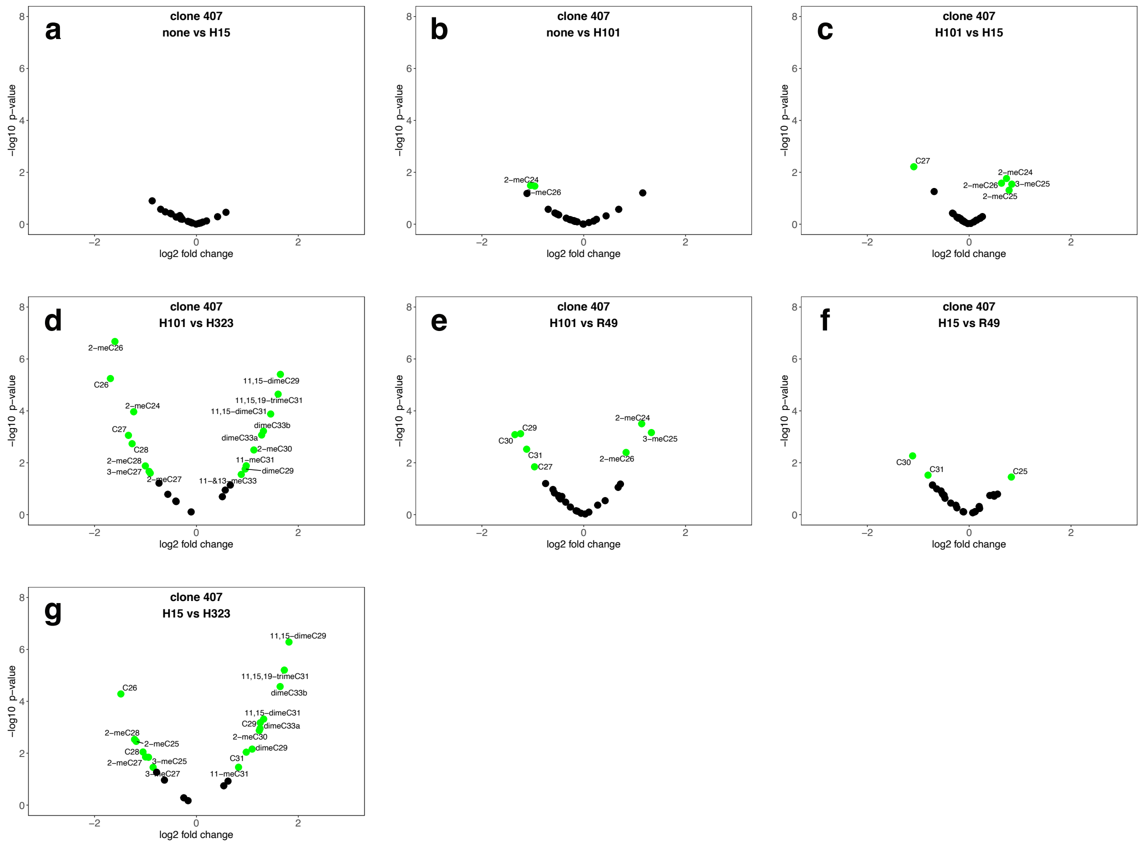

Figure S3 Volcano plots of clone 407 comparisons. (a & b) show differences between CHC profiles of uninfected and endosymbiont infected aphids. (c-g) show differences between CHC profiles of aphids with different endosymbiont infections. Points highlighted in green represent compounds with p-values <0.05 and log-fold changes >0.5.

405

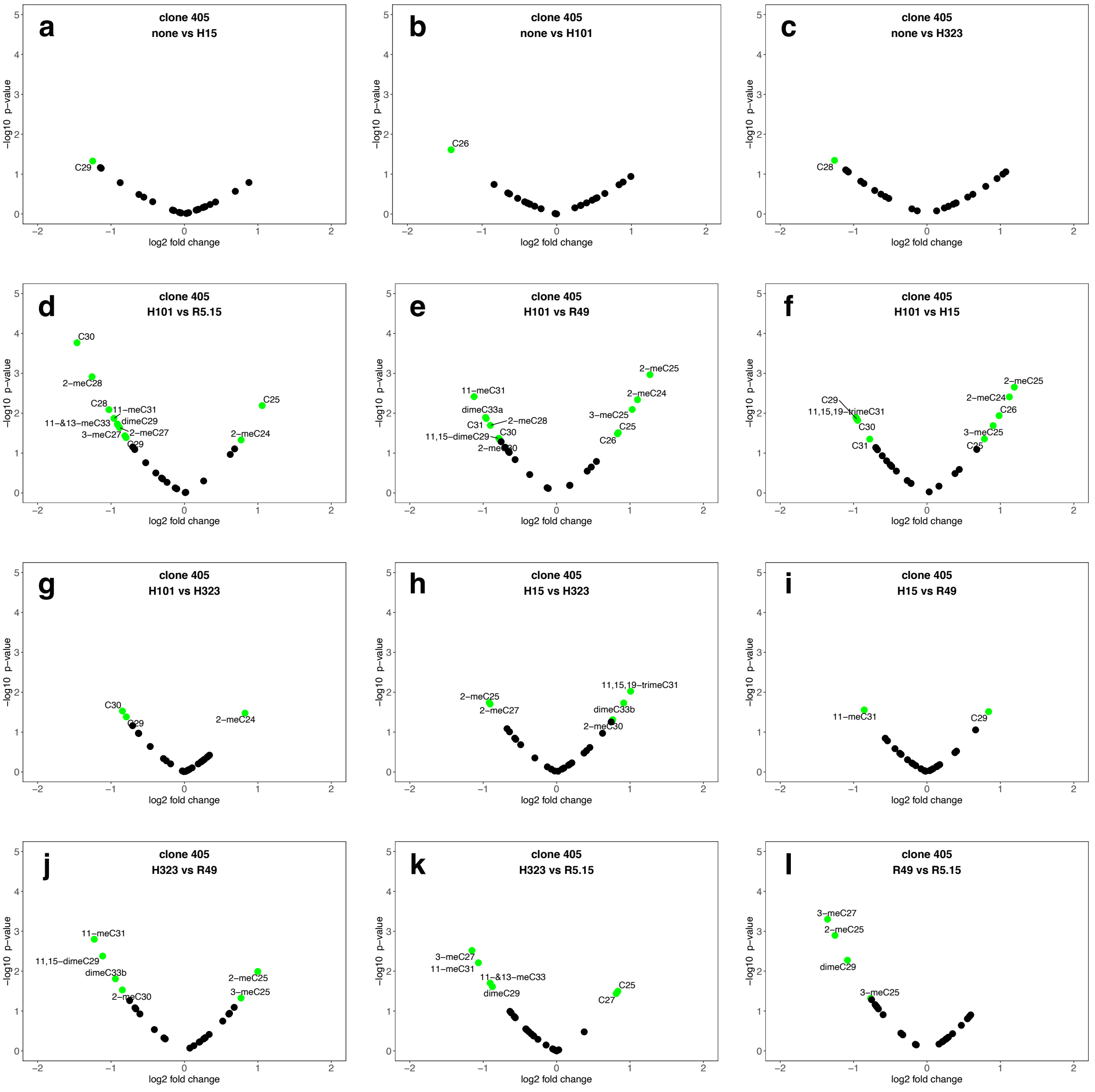

Figure S4 Volcano plots of clone 405 comparisons. (a, b, c) show differences between CHC profiles of uninfected and endosymbiont infected aphids. (d-l) show differences between CHC profiles of aphids with different endosymbiont infections. Points highlighted in green represent compounds with p-values <0.05 and log-fold changes >0.5.

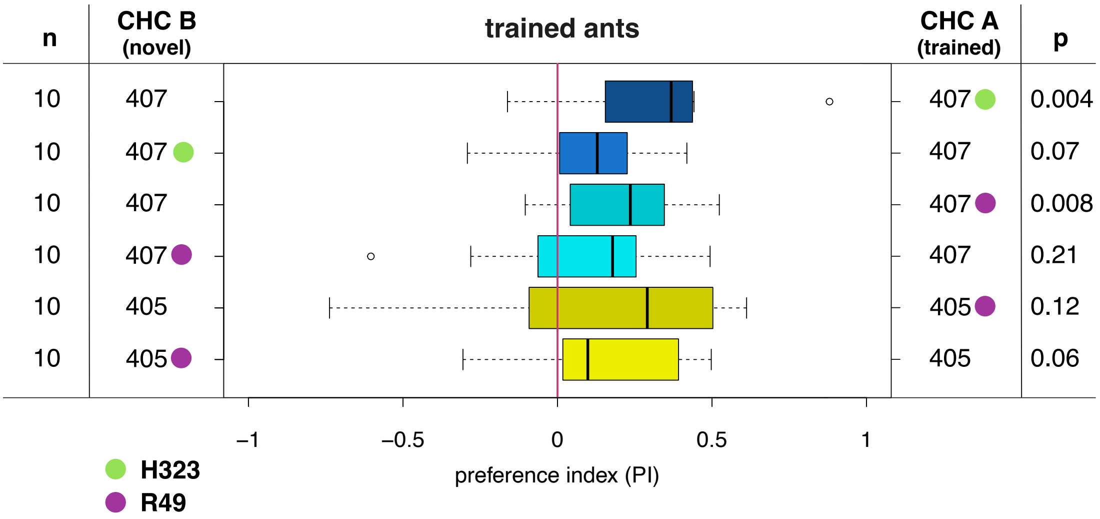

Figure S5 Preference indices (PI) of trained ants. Half of them were trained to the CHCs of uninfected aphids, the other half to CHCs of infected aphids. A PI of zero means no preference, a PI above or below zero a preference for CHC A (trained), or CHC B (novel), respectively. The number of replicates is indicated by n. Green dots represent an infection with H. defensa strain H323 and purple dots an infection with R. insecticola strain R49. shows the preference of trained ants.

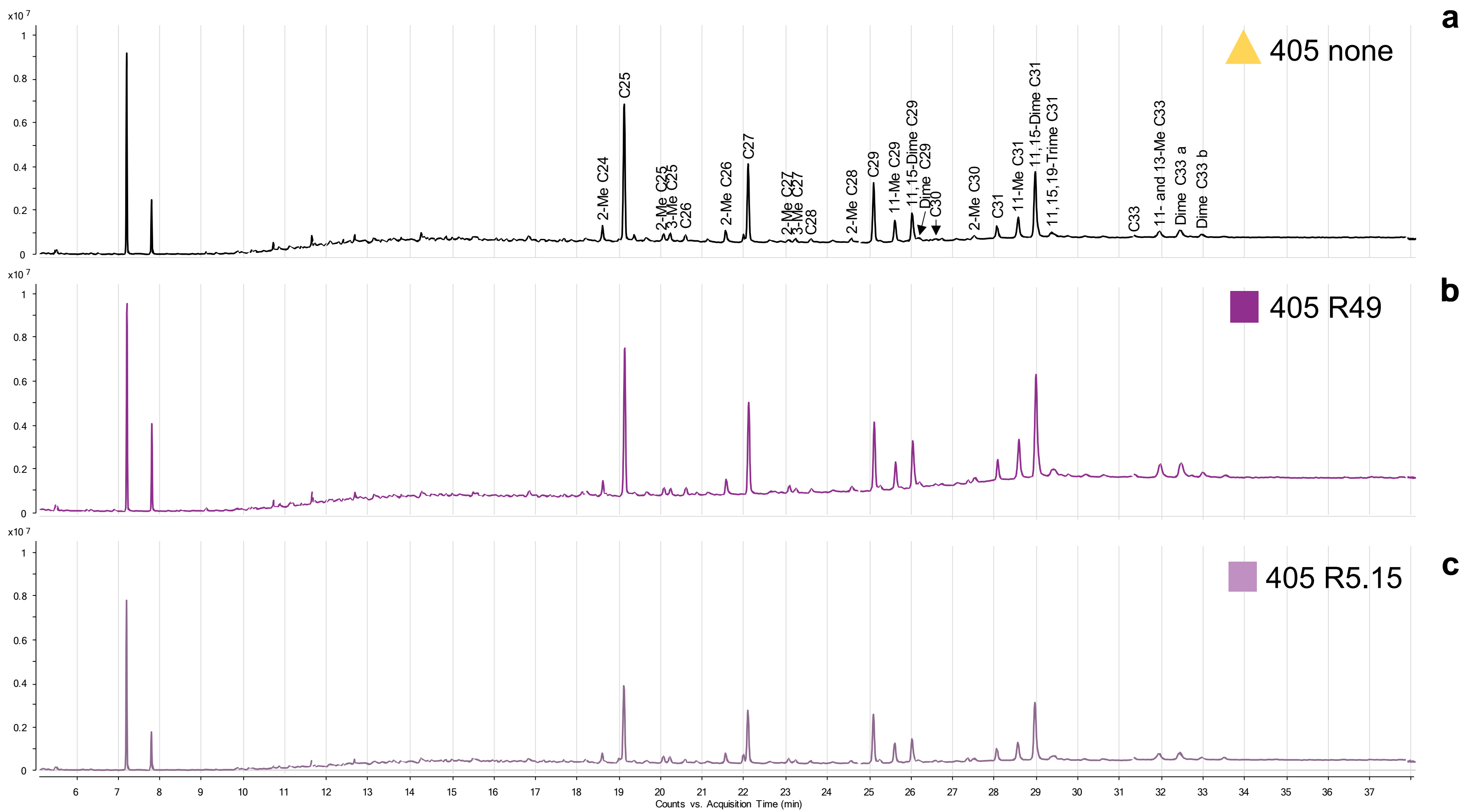

Figure S6 Gas chromatograms of cuticular hydrocarbons of (a) uninfected aphids of clone 405, (b) aphids of clone 405 infected with R. insecticola R49 and (c) aphids of clone 405 infected with R. insecticola R5.15. The peak labels in (a) also correspond to the peaks with the same retention times in (b) and (c).

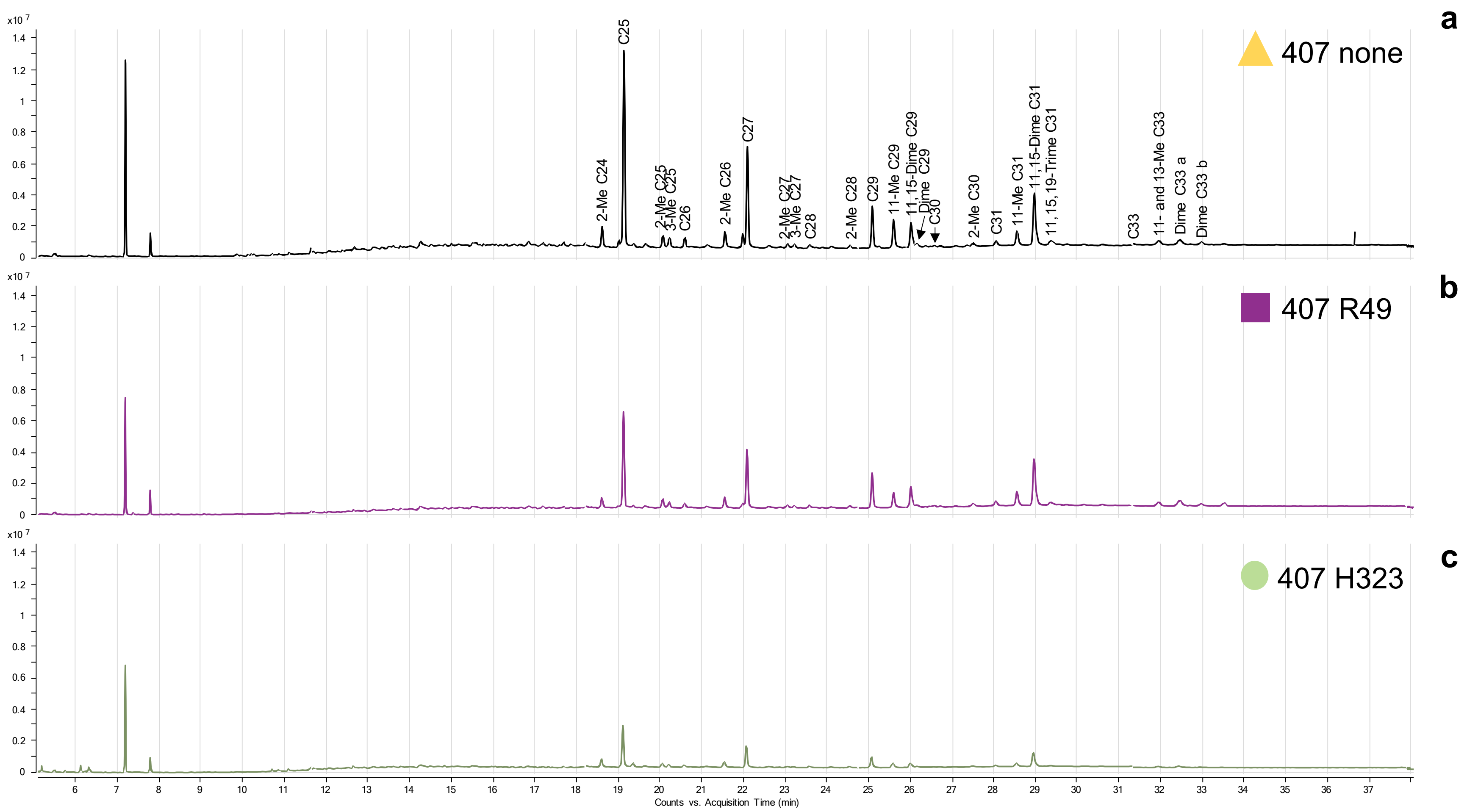

Figure S7 Gas chromatograms of cuticular hydrocarbons of (a) uninfected aphids of clone 407, (b) aphids of clone 407 infected with R. insecticola R49 and (c) aphids of clone 407 infected with H. defensa H323. The peak labels in (a) also correspond to the peaks with the same retention times in (b) and (c).
